# Characterization of lipid metabolism in a novel immortalized human hepatocyte cell line

**DOI:** 10.1152/ajpendo.00594.2014

**Published:** 2015-06-30

**Authors:** Charlotte J. Green, Deborah Johnson, Harsh D. Amin, Pamela Sivathondan, Michael A. Silva, Lai Mun Wang, Lara Stevanato, Catriona A. McNeil, Erik A. Miljan, John D. Sinden, Karl J. Morten, Leanne Hodson

**Affiliations:** ^1^Oxford Centre for Diabetes, Endocrinology and Metabolism, University of Oxford, Churchill Hospital, Oxford, United Kingdom;; ^2^Nuffield Department of Obstetrics and Gynaecology, The Women's Centre, University of Oxford, John Radcliffe Hospital, Oxford, United Kingdom;; ^3^ReNeuron Group, Guildford, Surrey, United Kingdom;; ^4^Department of Hepatobiliary and Pancreatic Surgery, Oxford University Hospital NHS Trust, Churchill Hospital, Oxford, United Kingdom; and; ^5^Department of Cellular Pathology, Oxford University Hospitals, Oxford, United Kingdom

**Keywords:** NAFLD, hepatocyte, *PNPLA3*, triglyceride, human

## Abstract

The development of hepatocyte cell models that represent fatty acid partitioning within the human liver would be beneficial for the study of the development and progression of nonalcoholic fatty liver disease (NAFLD). We sought to develop and characterize a novel human liver cell line (LIV0APOLY) to establish a model of lipid accumulation using a physiological mixture of fatty acids under low- and high-glucose conditions. LIV0APOLY cells were compared with a well-established cell line (HepG2) and, where possible, primary human hepatocytes. LIV0APOLY cells were found to proliferate and express some mature liver markers and were wild type for the *PNPLA3* (rs738409) gene, whereas HepG2 cells carried the Ile^148^Met variant that is positively associated with liver fat content. Intracellular triglyceride content was higher in HepG2 than in LIV0APOLY cells; exposure to high glucose and/or exogenous fatty acids increased intracellular triglyceride in both cell lines. Triglyceride concentrations in media were higher from LIV0APOLY compared with HepG2 cells. Culturing LIV0APOLY cells in high glucose increased a marker of endoplasmic reticulum stress and attenuated insulin-stimulated Akt phosphorylation whereas low glucose and exogenous fatty acids increased AMPK phosphorylation. Although LIV0APOLY cells and primary hepatocytes stored similar amounts of exogenous fatty acids as triglyceride, more exogenous fatty acids were partitioned toward oxidation in the LIV0APOLY cells than in primary hepatocytes. LIV0APOLY cells offer the potential to be a renewable cellular model for studying the effects of exogenous metabolic substrates on fatty acid partitioning; however, their usefulness as a model of lipoprotein metabolism needs to be further explored.

nonalcoholic fatty liver disease (NAFLD) is one of the most common liver diseases affecting approximately one-third of adults in the developed world who do not consume significant amounts of alcohol (9, 39); obesity is a well-documented risk factor (9). NAFLD encompasses a spectrum of conditions including steatosis [defined as intracellular lipid above 5% of hepatic tissue (16, 47)], steatohepatitis (NASH), and cirrhosis (8, 9, 11, 38, 39). To date, a variety of models including human, animal, and cell, have been used to investigate the mechanisms involved in the development and progression of NAFLD, as recently reviewed (20). However, to elucidate the causes and progression of NAFLD there is a need to develop a liver cell model that is not only liver-like and proliferative (providing a renewable alternative to human primary hepatocytes) but offers the opportunity to study processes that occur in a human liver in vivo, such as fatty acid partitioning (23).

The net retention of intracellular triglyceride (TG) (and cholesteryl esters) is the hallmark of hepatic steatosis (4), and a number of factors have been suggested to influence the accumulation of intrahepatocellular TG (20); ultimately fat accumulation represents an imbalance in fatty acid input and removal (23). Moreover, genotype has also been shown to be associated with liver fat content in humans (6, 44) and hepatic cell lines (3, 40) and therefore should be taken into consideration. The patatin-like phospholipase domain-containing 3 (*PNPLA3*) gene variant rs738409 (C>G polymorphism) is consistently demonstrated to be associated with liver fat content in humans (5); the genotype of human cell lines is not often reported (20). In vitro and cellular models provide the opportunity to investigate the molecular mechanisms involved in NAFLD development (20). Although primary human hepatocytes are considered the gold standard, they come with limitations including availability, donor variability (including genotype), being nonproliferative and therefore a finite source, and only able to be cultured for a limited time frame before loss of phenotype (7). Many hepatocyte/hepatocyte-like cell lines exist as alternative models to primary hepatocytes, and these offer advantages including the ability to proliferate and the relative ease of cryopreservation. The usefulness of many hepatocyte cell lines for investigations into the development of NAFLD are often hindered due to the cells not being well characterized in terms of the presence of key enzymes, response to metabolic nutrients, and mechanistic machinery required for TG synthesis/secretion and fatty acid oxidation.

Other factors that may impact on TG accumulation and fatty acid metabolism include culturing conditions (20). For example, both HepG2 and HepaRG cell lines have been shown to secrete TG-containing lipoproteins in a glucose-dependent manner (45, 52), and under high-glucose conditions immortalized human hepatocytes have been shown to increase lipoprotein secretion and lipogenic gene expression (45). High-glucose exposure is thought to contribute to endoplasmic reticulum (ER) stress through the unfolded protein response (18), which has been suggested to play a role in the development of lipid droplets and liver disease in many cell lines (13, 18, 56). The addition of exogenous fatty acids or insulin to culturing medium of liver cells appears to be undertaken by some, but not all; since both may affect fatty acid partitioning they are important factors to consider. The batch of serum used may also affect the secretion of lipids from cells (10). No standard culture conditions appear to be universally applied within and across studies (20), thus making interpretation across studies challenging.

Although available cell lines provide some insight into the development of NAFLD, it would be useful to develop cell models that are characterized in terms of culture conditions and the effect this may have on intracellular fatty acid partitioning. To address this, we set out to develop an immortalized human liver-derived cell line and use these cells to develop a model of TG accumulation by using a physiological mixture of exogenous fatty acids (representative of dietary fatty acids or nonesterified fatty acids from adipose tissue), under low- and high-glucose conditions. Effects of glucose on LIV0APOLY cells were compared with effects on HepG2 cells and, where possible, fatty acid partitioning was compared with primary human hepatocytes.

## METHODS

### 

#### Materials.

All reagents were obtained from Life Technologies (Paisley, Scotland) unless otherwise stated. Collagenase A, dispase II, and DNase I were purchased from Roche (Hertfordshire, UK). Fetal bovine serum (FBS) was purchased from HyClone (Northumberland, UK). Trypsin-EDTA was purchased from Lonza (Slough, UK). Normal goat serum (NGS) was purchased from Vector Labs (Peterborough, UK). Immunocytochemistry antibody against albumin was from Santa Cruz Biotechnology (Middlesex, UK). Western blot antibodies against cytokeratin 18 (CK18), α-fetoprotein (AFP), caspase-3, diacylglycerol acyltransferase-2 (DGAT2), and β-actin were from Abcam (Cambridge, UK), anti-albumin (human) was from Universal Biologicals (Cambridge, UK), anti-AMPK Thr^172^, anti-Akt Ser^473^, anti-Akt, and anti-CCAAT/enhancer-binding protein homologous protein (CHOP) were from Cell Signaling (Danvers, MA), and anti-apoB was from Santa Cruz Biotechnology (Santa Cruz, CA). Anti-AMP-activated protein kinase (AMPK)α1 and -α2 were a gift from Prof. Grahame Hardie (Dundee, UK). The bicinchoninic acid (BCA) protein assay was from Thermo Fischer Scientific (Northumberland, UK). Peroxidise-conjugated IgG antibodies were from Dako (Cambridge, UK). Urea, 3-hydroxybutyrate (3-OHB), and TG assays were from Instrumentation Laboratory UK (Cheshire, UK). Collagenase IV, calcium chloride, and Hanks' balanced salt solution (HBSS) were purchased from Sigma-Aldrich (Dorset, UK).

#### Derivation of cell line.

Human fetal liver tissue (15 wk gestation) was obtained from Advanced Bioscience Resources (Alameda, CA) following normal terminations and in accordance with nationally (UK and USA) approved ethical and legal guidelines. Maternal blood was screened and found to be free of infectious agents. Fetal liver tissue was washed twice in calcium-free HEPES, pH 7.2, 4°C, once in calcium-free HEPES, pH 7.2, 4°C with 0.5 mM EGTA, and then diced using scalpels. Tissue was incubated at 37°C for 20 min in digestion buffer (calcium-free HBSS, 0.05% collagenase A, 0.005% DNase I, 0.0125% hylaronidase, 0.025% dispase II, 5 mM calcium chloride). Cell suspension was filtered through a sterile nylon mesh and collected in isolation medium [DMEM, 10% FBS, 50 mg/ml gentamicin, 2 mM l-glutamine, 20 ng/ml epidermal growth factor (EGF)]. Cell suspension was washed three times by pelleting the cells at 50 g and the supernatant discarded each time. The cells were plated on collagen-coated plates in isolation medium and left to attach for 4 h. Cells were washed once with phosphate-buffered saline (PBS; 37°C) before addition of hepatocyte selection medium [arginine-free Williams E medium, 2.2 g/l sodium bicarbonate, 10% FBS, 2 mM l-glutamine, 100 nM insulin, 100 U/ml penicillin-streptomycin (PS), 0.4 mM l-ornithine, 5.5 μM hydrocortisone, 20 ng/ml EGF; 20 ng/ml]. Cells were infected with MMLV retrovirus containing the c-mycER^TAM^ gene as previously described (42). From this stage, cells were grown in hepatocyte growth medium [DMEM (22.5 mM glucose) + Glutamax, 10% FBS, 1% nonessential amino acids (NEAA), 1% PS] containing 100 nM tamoxifen (4-OHT). Selection for cells expressing the c-mycER^TAM^ construct was carried out by incubation with 25 μg/ml geneticin for 14 days. Resulting cells (LIV0APOLY) were cryopreserved in FBS containing 10% dimethyl sulfoxide.

#### Isolation of human hepatocytes.

Liver was obtained from patients undergoing surgery who had consented (NRES Committee South Central; Berkshire B 11/SC/0443) to the use of excess tissue (resection surplus) for research. The resection surplus (collected on ice) was examined and cut up by a pathologist, and only tissue deemed healthy (≥25 g) were used for hepatocyte isolation. Under aseptic conditions, tissue was diced using two scalpels and washed in HBSS in a stainless steel tea strainer to remove excess blood. Tissue was transferred to a specimen container containing prewarmed EGTA buffer (HBSS, 0.5 mM EGTA, 0.5% fatty acid-free BSA) and agitated (100 rpm) for 10 min at 37°C. Tissue was then placed in the tea strainer and washed three times in HBSS to remove blood and EGTA, and then placed in prewarmed digestion buffer (HBSS, 0.05% collagenase IV, 0.5% fatty acid-free BSA, 10 mM CaCl_2_) and agitated (100 rpm) for 30 min at 37°C. Digested tissue was passed through the tea strainer and supernatant collected and strained through a 100-μm cell strainer and kept on ice; the remaining tissue was again digested in fresh buffer. Collected supernatant was pooled and centrifuged (80 *g*, 5 min, 4°C). The supernatant was then discarded and hepatocyte pellet gently resuspended. Red blood cell lysis buffer (RBCly) was added to the hepatocyte suspension and left at room temperature for 3 min before inactivation with PBS. Cells were then centrifuged (80 *g*, 5 min, 4°C). Hepatocytes were washed twice in William's E buffer, and cells were counted and viability (mean 78%) was measured by Trypan blue exclusion. Cells were diluted to 1 million cells per millliliter in William's E buffer containing hepatocyte supplements.

#### Cell culture.

LIV0APOLY cells were grown in hepatocyte growth medium [DMEM (22.5 mM glucose) + Glutamax, 10% FBS, 1% NEAA, 1% PS] containing 100 nM tamoxifen (4-OHT). Medium containing 4-OHT was changed every 48 h. Cells were passaged when they reached 80–90% confluence by using trypsin-EDTA. Experiments were carried out on proliferating, confluent, or differentiated cells. Differentiation was achieved by first growing the cells to confluence (∼4 days) on collagen-coated plates in hepatocyte growth medium (with 4-OHT) and then arresting growth by changing the medium to that without the 4-OHT for 48 h. HepG2 cells were cultured in DMEM supplemented with 10% FBS, 1% NEAA and 1% penicillin-streptomycin. All experiments were performed on cells between passages 9 and 25. Primary human hepatocytes were plated in type I collagen-coated 12-well plates in William's E buffer containing hepatocyte supplements and 2% human serum (HS) and left to adhere for 4 h following isolation. After this, human hepatocytes were washed and cultured for a further 24 h in William's E buffer for experiments.

#### Fatty acid treatments.

LIV0APOLY cells were grown for 48 h on low-glucose (LG, 5.5 mM) or high-glucose (HG, 22.5 mM) DMEM in the absence of 4-OHT. After 24 h of glucose treatment, cells were treated with 0.25% fatty acid-free BSA alone or conjugated to 49.5 μM oleic, 33 μM palmitic, and 27.5 μM linoleic acid (OPL; physiological ratio 45:30:25%) in the absence or presence of 100 nM insulin for 24 h. Adherent primary hepatocytes were cultured in William's E buffer (11 mM glucose) supplemented with 0.25% fatty acid-free BSA alone or conjugated to OPL, as described above, for 24 h.

#### Cell growth measurements.

Proliferation was measured with a CyQUANT fluorescence assay according to the manufacturer's instructions.

#### Immunocytochemistry.

Cells were washed twice in PBS and then fixed for 15 min in 4% paraformaldehyde. Cells were permeabilized with 0.1% Triton X-100 for 10 min, and nonspecific binding was blocked with 10% NGS for 1 h at room temperature. Cells were incubated with primary antibodies for 2 h at room temperature. After washing twice with PBS, they were incubated for 1 h at room temperature with the appropriate Alexa dye-conjugated secondary antibody, and nuclei were counterstained with 10 mM Hoechst 33342 for 3 min.

#### Absolute quantification of cMycER^TAM^ copy number by quantitative real time PCR.

Total RNA from LIV0APOLY cells was isolated using a miRNeasy (Qiagen) kit according to the manufacturer's instructions. Total RNAs were reverse-transcribed into first-strand cDNA using a mix of random primer and poly(dT) and superscript II reverse transcriptase kit (Invitrogen) according to the manufacturer's instructions. qRT-PCR primers (forward agaggagcccagccagac, reverse tgtaaggaatgtgctgaagtgg, and Universal Probe Library 77) were designed specifically to detect the cMycER^TAM^ transgene using the Lightcycler LC480 system (Roche). To carry out absolute quantification of c-MycER^TAM^ copy numbers, a serial dilution of a known amount of cMycER^TAM^ plasmid was used to plot a standard curve. Copy numbers were calculated per 1 ng of retrotranscribed total RNAs.

#### Quantitative real-time PCR.

First-strand cDNA was synthesized from 0.5 μg of total RNA using a High Capacity Reverse Transcription kit. SYBER Green qRT-PCR reactions were performed in triplicate using a 1:10 dilution of cDNA with custom-designed primers [HRPT1 (hypoxanthine phosphoribosyltransferase-1): forward TTGCTTTCCTTGGTCAGGCA, reverse ATCCAACACTTCGTGGGGTC; HNF1 (hepatocyte nuclear factor 1): forward TCCTTCCAGCTAGTGACCCA, reverse CAGGCTCTGGCACAGAGTAG]. Reactions were carried out using a Rotor-Gene 6000 machine. TAQMAN qRT-PCR reactions were performed in triplicate using a 1:30 dilution of cDNA and Applied Biosystems assays (Table 1) and KAPA Probe Fast Master Mix (KAPA Biosystems). Reactions were run on an Applied Biosystems 7900HT machine. To adjust for variations in the cDNA synthesis, each gene was normalized to that of hypoxanthine-guanine phosphoribosyltransferase-1 (HPRT1) and or GAPDH mRNA using the comparative (ΔΔC_T_) method (41).

**Table 1.** Taqman assays

Gene	ABI Assay ID
*ACC2*	Hs00153715_m1
*PNPLA2*	Hs00386101_m1
*ACC1*	Hs01046047_m1
*FASN*	Hs01005622_m1
*GAPDH*	Hs02758991_g1
*HRPT1*	Hs02800695_m1

#### Genotyping.

*PNPLA3* rs738409 genotype was determined by allelic discrimination using TaqMan reagents (assay ID: C724110, Applied Biosystems) according to the manufacturer's protocol.

#### Immunoblotting.

Protein concentrations were determined using the BCA protein assay. Briefly, 15–20 μg of whole cell lysates were subjected to SDS-PAGE using NOVEX 4–20% precast gels. Polyvinylidene difluoride (PVDF) membranes were probed with primary antibodies raised against the protein of interest, as indicated in the figure legends. Detection of primary antibodies was performed using appropriate peroxidase-conjugated IgG, and protein signals were visualized using enhanced chemiluminescence and exposure to autoradiographic film. Quantification of immunoblots was done using Image J software (NIH, Bethesda, MD; http://rsb.info.nih.gov/ij). For apoB, the medium collected from cells that were incubated with lipoprotein-deficient serum in the absence or presence of fatty acids (OPL) was concentrated using Amicon Ultra Centrifugal Filter units (Millipore, Heretfordshire, UK) at 3,750 *g* for 15 min using a swing-out rotor followed by centrifugation at 1,000 *g* for 2 min to recover the sample. Ten microliters of concentrated sample was subjected to SDS-PAGE (as above). Medium containing BSA only was treated in the same way as experimental samples as a negative control.

#### Stable isotope-labeled fatty acids ([D_31_]- and [U-^13^C]palmitate) culturing and analysis.

To trace the fate of the exogenous fatty acids, LIV0APOLY cells were cultured in 15% lipoprotein-deficient FBS (Sigma-Aldrich, Dorset, UK), and hepatocytes were cultured in serum-free medium in the presence of 0.25% fatty acid-free BSA conjugated to OPL, where the palmitate was labeled [50% D_31_, 50% U-^13^C (CK Gas, Cambridgeshire, UK)] for 24 h, and medium and cells were collected for analysis. An internal standard was added, prior to the extraction of total lipids (17), for quantification of TG being extracted from cells and cell medium. Fatty acid methyl esters (FAMEs) from TG were prepared and analyzed by gas chromatography (GC) as described previously (12). The addition of the stable isotope-labeled fatty acids was used to distinguish exogenous from de novo lipogenesis (DNL)-derived fatty acids in TG. [D_31_]- and [U-^13^C]palmitate enrichments were determined simultaneously by GC-mass spectrometry (GC-MS) using a 5890 GC coupled to a 5973N MSD (Agilent Technologies UK). Ions with mass-to-charge ratios (*m/z*) of 270 (M+0), 301 (M+31), and 286 (M+16*)* were determined by selected ion monitoring. Tracer-to-tracee ratios (TTRs) for [D_31_]palmitate (M+31)/(M+0) and [U-^13^C]palmitate (M+16)/(M+0) were multiplied by the corresponding palmitate-TG concentrations to give tracer concentrations from which the relative (%) contribution of exogenous fatty acids to total TG was calculated.

As a marker of fatty acid oxidation, we measured the appearance of ^2^H_2_O using a Finnigan GasBench-II (ThermoFisher Scientific, UK) in the medium that would have been derived from the [D_31_]palmitate added to the culture medium (29, 51).

#### Urea, total TG, and 3-OHB concentrations.

Urea and TG were analyzed using Instrumentation Laboratory kits on an ILab 650 Clinical Chemistry analyzer. The TG method was adapted to enable analysis of low concentrations, and 3-OHB was analyzed as previously described (35). Serum-free and medium-containing FBS or lysis buffer were used as background controls.

#### Oil red O staining.

Cells were washed twice in PBS and fixed using 10% formalin for 1 h. Cells were then washed in 60% isopropanol and left to dry. Staining was carried out by adding Oil Red O (6 mM; Sigma-Aldrich, Dorset, UK) for 1 h at room temperature. Cells were then counterstained using H & E stain and mounted onto slides using aquamount.

#### Statistics.

For multiple comparisons, statistical analysis was performed using one-way or two-way analysis of variance (ANOVA) with Bonferoni corrections. Data analysis was performed using GraphPad Prism software and considered statistically significant at *P* < 0.05.

## RESULTS

### 

#### Growth and differentiation properties of LIV0APOLY cells.

**Fig. 1.**
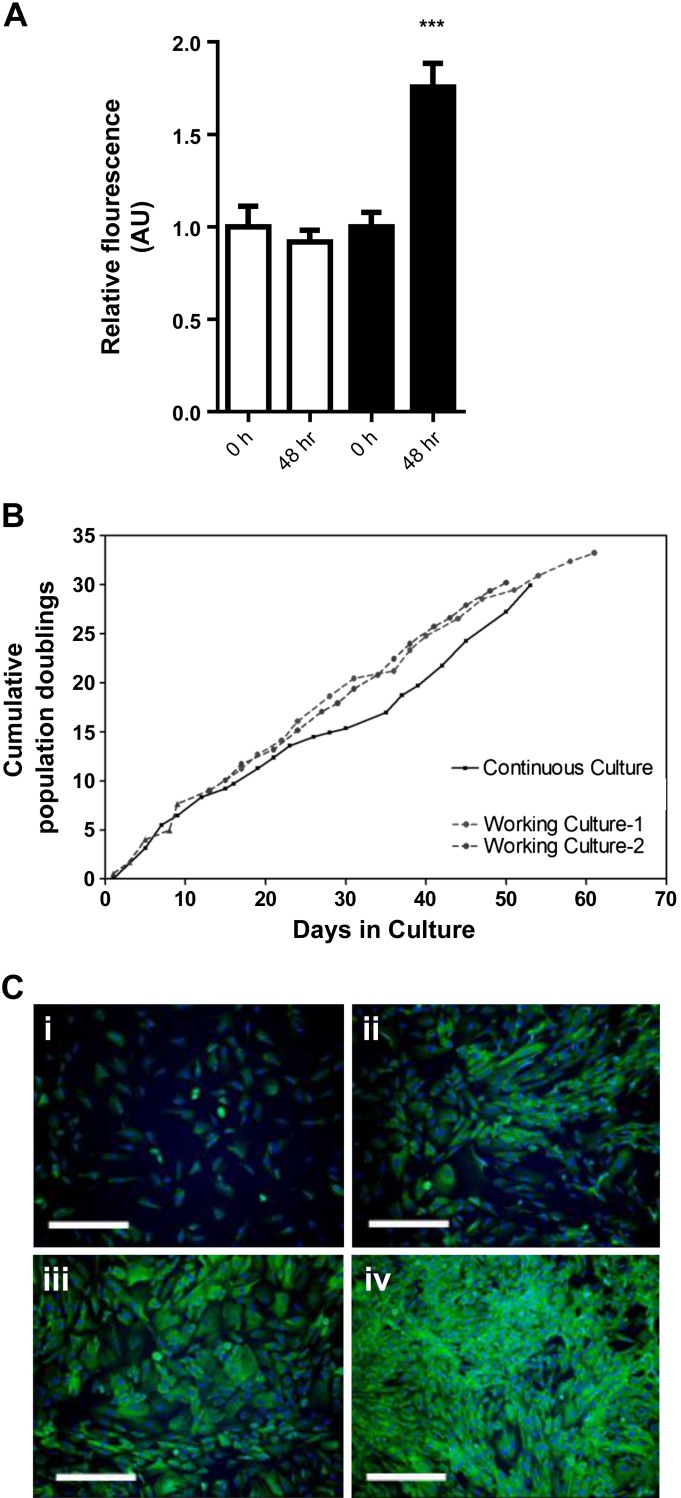
Growth characterization of the novel human liver cell line LIV0APOLY. *A*: CYQUANT analysis of cell growth (*n* = 4). *B*: cumulative population doublings of LIV0APOLY cells that had been cryopreserved, thawed, and passaged 3 times [working cultures 1 and 2 (dotted line, ●)] compared with LIV0APOLY cells that were continuously cultured and not cryopreserved (solid line, ■). *C*: imunoflourescent staining for albumin (×10 objective; scale bar, 50 μm) in LIV0APOLY cells seeded at different densities: *i*) 2,000, *ii*) 5,000, *iii*) 10,000, *iv*) 15,000. Data are means ± SE. ^***^P < 0.001, Time 0 h vs. 48 h.

Expression of c-mycER^TAM^ was confirmed by using a set of primers designed to amplify a unique coding sequence of the cMycER^TAM^ construct (expression 1.42 × 10^5^ ± 0.057 × 10^5^; data not shown). Growth of LIV0APOLY cells was dependent on the presence of 4-OHT in the medium (Fig. 1*A*). Cells had a population doubling time of ∼48 h and were capable of at least 30 population doublings in culture over 60 days (Fig. 1*B*, continuous culture). LIV0APOLY cells were cryopreserved three times to create consecutive cell “banks”; this did not adversely affect the population doubling capacity (Fig. 1*B*, working cultures 1 and 2). Proliferating LIV0APOLY cells expressed low amounts of albumin, which dramatically increased when cells were confluent (Fig. 1*C*, *i–iv*), and immunofluorescent staining of proliferating cells demonstrated consistent expression of albumin over an extended period of culture (≥45 days; data not shown).

#### Phenotype of differentiated LIV0APOLY cells is dependent on culture time.

**Fig. 2.**
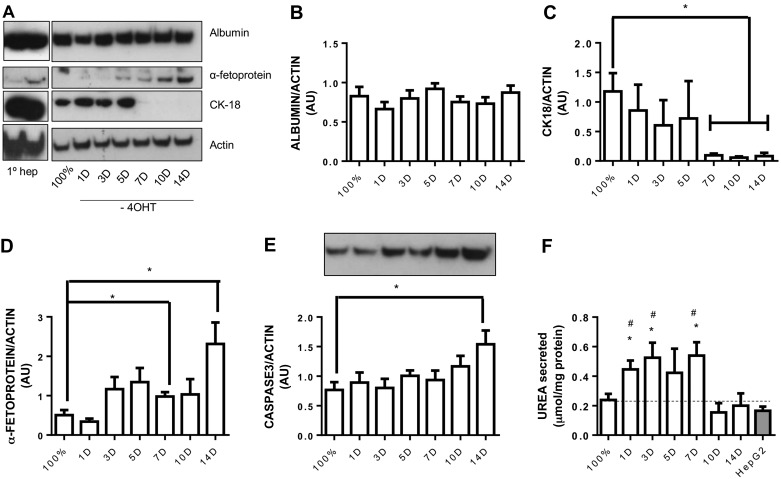
Differentiation time course in LIV0APOLY cells. *A*: immunoblots of albumin, α-fetoprotein, and cytokeratin (CK)18, equal gel loading ascertained by immunoblotting against β-actin. Primary hepatocytes (promocell, 10 μg) were immunoblotted as control for antibodies. Expressions of albumin (*B*), CK18 (*C*), α-fetoprotein (*D*), and caspase-3 (*E*) were quantified relative to β-actin (*n* = 4) and expressed as arbitrary units (AU). *F*: Urea secreted from LIV0APOLY and HepG2 cells over 24 h into cell culture medium (*n* = 4). *G*: Data are means ± SE. *P < 0.05 significantly different from confluent cells (100%); #*P* < 0.05 significantly different from HepG2 cells.

To further characterize LIV0APOLY cells, the expression of human liver markers were measured in fully confluent cells over a time course in the absence of 4-OHT. Albumin protein expression remained constant when 4-OHT was withdrawn (up to at least 14 days in culture; Fig. 2, *A* and *B*). Cells cultured for up to 5 days without 4-OHT had comparable expression of cytokeratin 18 (CK18) to that of fully confluent cells; however, after 7 days in culture without 4-OHT, CK18 levels were significantly reduced (Fig. 2, *A* and *C*). Expression of AFP increased in cells after 7–14 days without 4-OHT (Fig. 2, *A* and *D*). Total caspase-3 expression was significantly elevated 14 days after removal of 4-OHT (Fig. 2*E*). Proliferating, fully confluent, or differentiated cells did not express CK19, measured by immunofluorescence or Western blot (data not shown). The amount of urea in the medium was significantly elevated from LIV0APOLY cells that were lacking 4-OHT for 1–3 days compared with fully confluent cells (Fig. 2*F*). Compared with the well-utilized cell line HepG2, there was significantly more urea in the medium of differentiated (1–7 days after 4-OHT removal) LIV0APOLY cells (Fig. 2*F*).

#### Differentiated phenotype is unaffected by glucose concentration in culture medium.

**Fig. 3.**
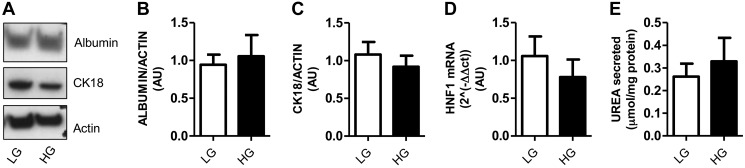
Effect of glucose concentration on expression of liver markers in LIV0APOLY cells. Cells were cultured under low-glucose (LG; 5.5 mM) or high-glucose (HG; 22.5 mM) conditions. *A*: immunoblots of albumin and CK18, equal gel loading ascertained by immunoblotting with an antibody against β-actin. Expression of albumin (*B*) and CK18 (*C*) were quantified relative to β-actin (*n* = 3) and expressed as AU. *D*: expression of *HNF1α* (hepatic nuclear factor 1α) was measured (*n* = 3). Housekeeping gene used was *HPRT1* (hypoxanthine-guanine phosphoribosyltransferase). *E*: amount of urea in cell culture medium over 24 h (*n* = 3). Data are means ± SE.

Many studies appear to culture hepatocyte cell lines in high-glucose (22.5 mM) medium however, this is not a physiological concentration in healthy humans, even in the postprandial state (36). All previous data characterizing the LIV0APOLY cells were carried out under high-glucose (HG) conditions; therefore, we investigated whether a lower-glucose (5.5 mM) concentration had an effect on the differentiated phenotype of LIV0APOLY cells (2 days postconfluence, without 4-OHT). Glucose concentration had no effect on protein expression of albumin or CK18 (Fig. 3, *A–C*). Additionally, levels of HNF1α mRNA were equally expressed at both glucose concentrations (Fig. 3*D*), and concentration of urea in medium was also unaffected by glucose concentration (Fig. 3*E*).

#### Effect of glucose and PNPLA3 genotype on TG accumulation and secretion.

**Fig. 4.**
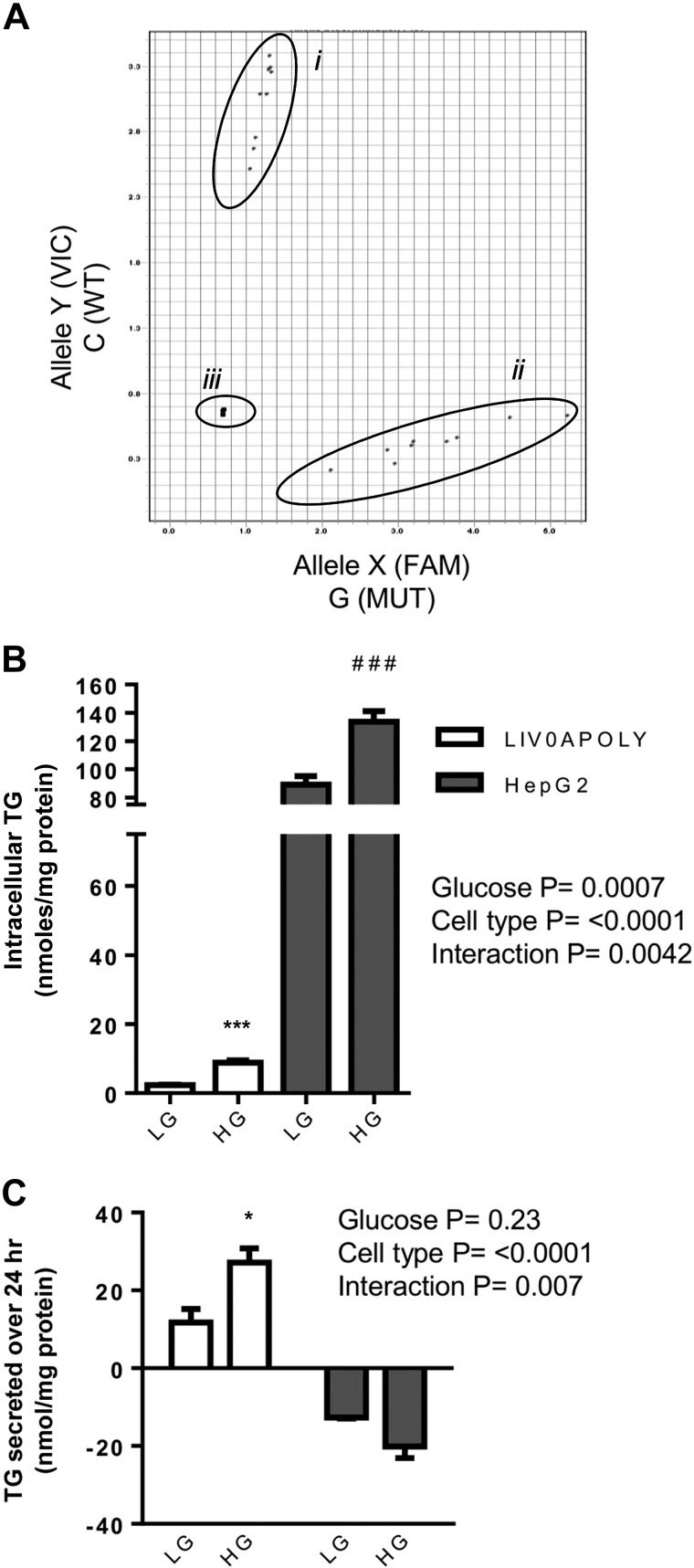
*PNPLA3* genotype and triglyceride (TG) profile of LIV0APOLY and HepG2 cells under HG and LG conditions. *A*: *PNPLA3* rs738409 genotype *i* = LIV0APOLY, *ii* = HepG2, and *iii* = no template control. *x*-Axis represents wild-type allele; *y*-axis mutant allele. TG contents of cells (*B*) and media (*C*) (24 h) were measured (*n* = 3) in cells cultured in LG (5.5 mM) or HG (22.5 mM) media. Data are means ± SE. *P < 0.05, ***P < 0.001 LG vs. HG in LIV0APOLY cells; ###*P* < 0.001 LG vs. HG in HepG2 cells.

Information regarding the *PNPLA3* genotype of liver cell lines is not widely reported (20). We found LIV0APOLY cells were homozygous wild-type (CC) for *PNPLA3*, whereas HepG2 cells carry the rs738409 variant (GG) (Fig. 4*A*). Consistent with genotype, HepG2 cells had significantly higher intracellular TG content than LIV0APOLY cells [111.5 ± 26.5 vs. 5.6 ± 3.6 nmol/mg protein (mean ± SE), respectively; Fig. 4*B*]. Since excess glucose may also promote liver fat accumulation (15), we investigated how glucose concentration affected TG accumulation and secretion in both LIV0APOLY and HepG2 cell lines. Exposure to HG significantly increased the amount of intracellular TG in both cell lines compared with LG (Fig. 4*B*). To establish whether cells secreted TG, we measured the presence of TG in medium from LIV0APOLY and HepG2 cells. We found that the medium from LIV0APOLY cells contained higher than background amounts of TG and that this was increased in HG compared with LG (Fig. 4*C*). Conversely, HepG2 cells had undetectable amounts of TG in the medium with either glucose concentration (Fig. 4*C*).

#### Effects of treatment of LIV0APOLY cells with exogenous fatty acids and insulin under HG and LG conditions.

**Fig. 5.**
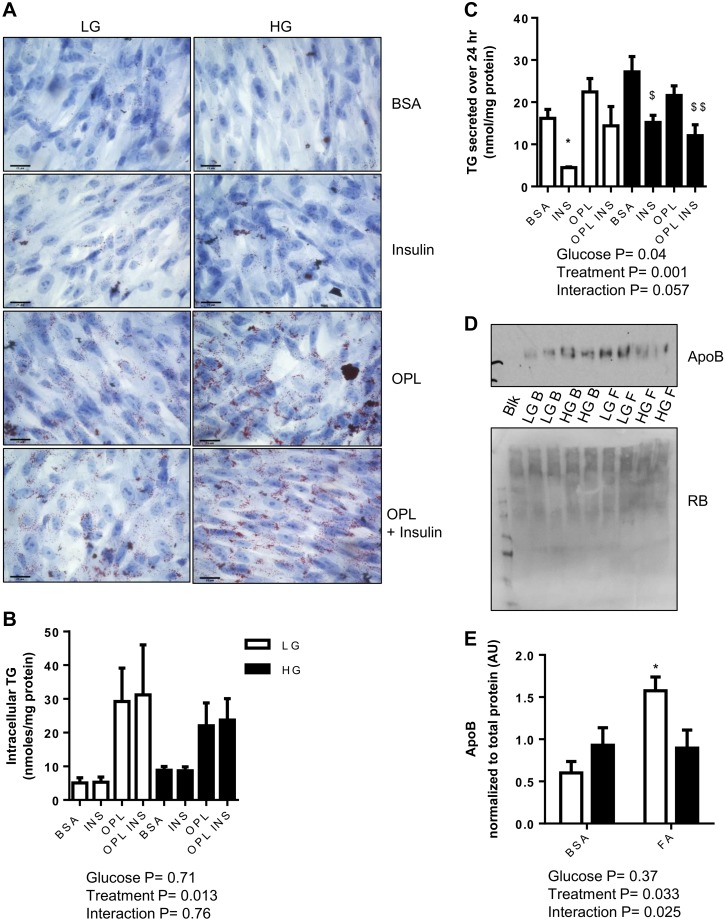
Neutral lipid accumulation and secretion in LIV0APOLY cells under HG and LG conditions. Cells were cultured under LG (5.5 mM) or HG (22.5 mM) and treated with insulin (INS; 100 nM) in the absence or presence of OPL (50 μM oleic, 33 μM palmitic, and 27 μM linoleic acid) for 24 h. *A*: representative images (×40 objective) of neutral lipid, scale bar 25 μm. Total TG of cells (*B*) and in media (*C*) (24 h). *D*: immunoblot of apoB in media and reactive brown (RB, total protein; Blk, blank; B, BSA; F, OPL). *E*: expression of apoB was quantified relative to protein concentration. Data are means ± SE (*n* = 3). *P < 0.05 significantly different from LG-BSA; $*P* < 0.05, $$*P* < 0.01 significantly different from HG-BSA.

Oil Red O stain was used to visualize neutral lipid (TG and cholesteryl ester) within the cells (37, 43). LIV0APOLY cells grown in HG or LG with or without fatty acids (OPL) in the absence or presence of insulin accumulated neutral lipid (Fig. 5*A*). Biochemical quantification of intracellular TG showed that in both glucose conditions TG was significantly increased in cells treated with fatty acids with or without insulin (Fig. 5*B*). The addition of insulin significantly reduced the amount of TG in the medium from LIV0APOLY cells in the absence or presence of exogenous fatty acids (Fig. 5*C*). However, addition of exogenous fatty acids alone did not increase the concentration of TG in the medium under either glucose condition (Fig. 5*C*). Although low, the appearance of apoB in the medium from LIV0APOLY cells was significantly increased when cells were treated with OPL under LG conditions (Fig. 5, *D* and *E*). The effect of HG on TG in the medium were less obvious and more variable; insulin appeared to suppress TG secretion under HG conditions (Fig. 5*C*, filled bars), but there was no detectable effect of OPL treatment. Treatment with OPL did not significantly increase apoB in the medium under HG conditions (Fig. 5, *D* and *E*).

#### Effect of exogenous fatty acids and glucose concentration on expression of TG synthesis enzymes DGAT1/2 in LIV0APOLY cells.

**Fig. 6.**
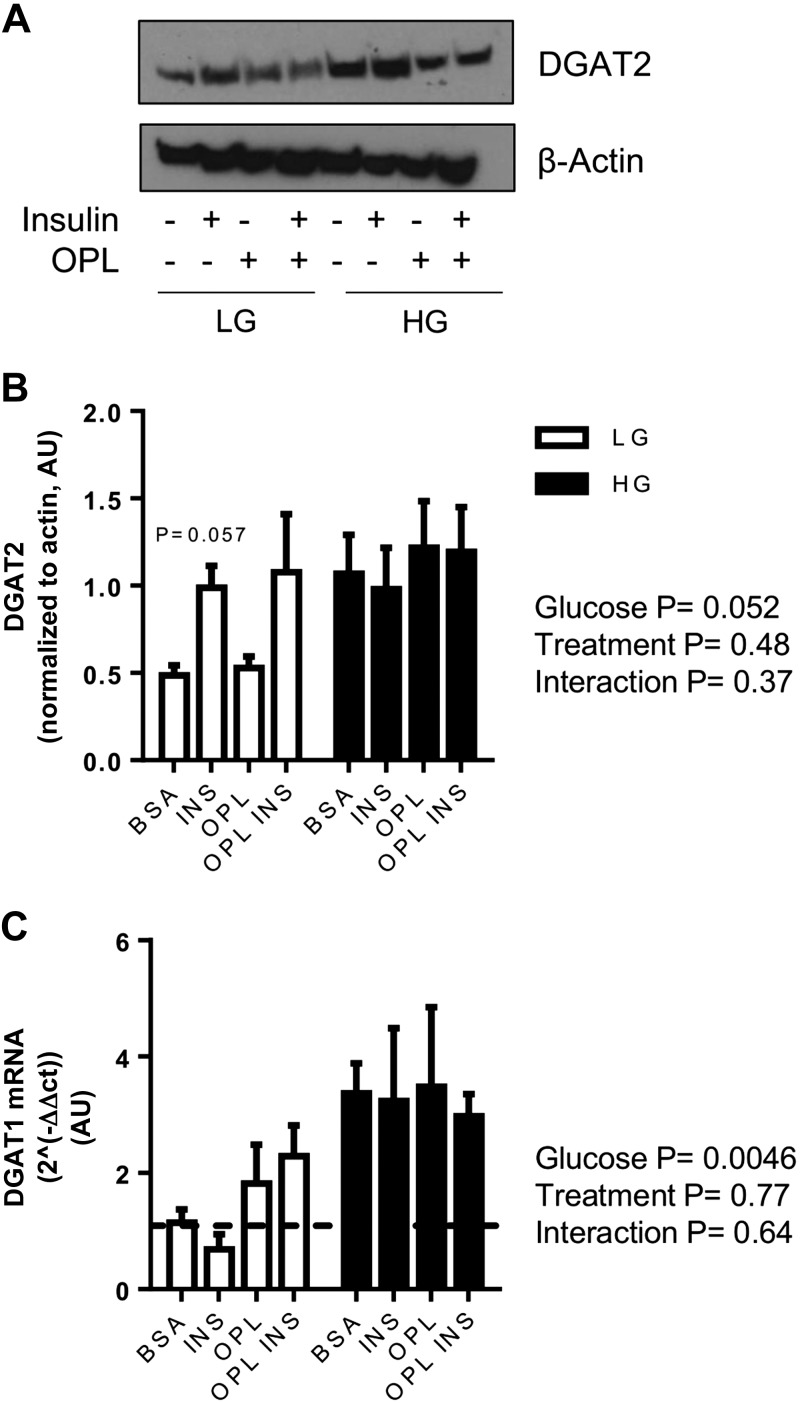
Effect of glucose and exogenous fatty acids on diacylglycerol acyltransferases (DGATs). Cells were cultured under LG (5.5 mM) or HG (22.5 mM) and treated with insulin (100 nM) in the absence or presence of OPL for 24 h. *A*: immunoblot of DGAT2. Equal gel loading was ascertained by immunoblotting against β-actin. *B*: expression of DGAT2 quantified relative to β-actin (*n* = 3) and expressed as AU. *C*: mRNA expression of DGAT1 (*n* = 3). Housekeeping gene was *HPRT1*. Data are means ± SE.

Culturing cells in HG resulted in significantly elevated DGAT2 protein (Fig. 6, *A* and *B*) and DGAT1 mRNA (Fig. 6*C*) expression. Under LG conditions, insulin robustly increased DGAT2 protein expression (*P* = 0.057; Fig. 6, *A* and *B*). Lipid loading (OPL) had no significant effect on DGAT1 mRNA or DGAT2 protein expressions (Fig. 6, *A–C*). However, under LG conditions there was a trend toward OPL treatment increasing the expression of both DGAT1 and DGAT2 when insulin was absent.

#### Effect of exogenous fatty acids and glucose concentration on insulin signaling and ER stress in LIV0APOLY cells.

**Fig. 7.**
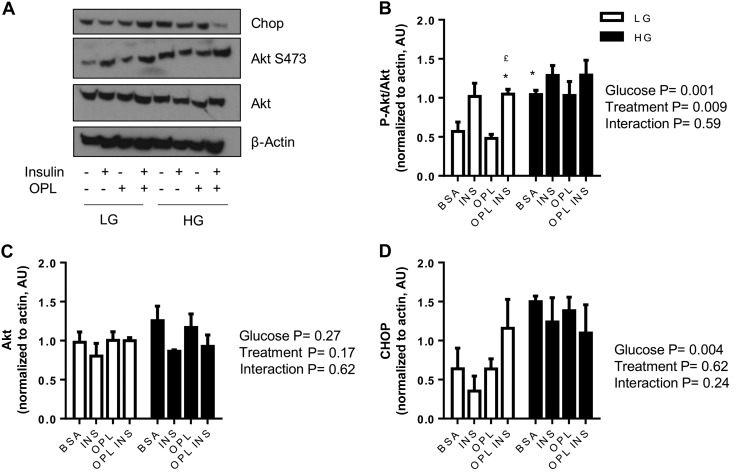
Effect of glucose and exogenous fatty acids on insulin signaling and endoplasmic reticulum (ER) stress in LIV0APOLY cells. Cells were cultured under LG (5.5 mM) or HG (22.5 mM) and treated with insulin (100 nM) in the absence or presence of OPL for 24 h. Immunoblots of CCAAT/enhancer-binding protein homologous protein (CHOP), Akt, and Ser^473^ Akt, equal gel loading ascertained by immunoblotting against β-actin. Expressions of Akt Ser^473^ (*B*), Akt (*C*), and CHOP (*D*) were quantified relative to β-actin (*n* = 3) and expressed as AU. Data are means ± SE. *P < 0.05 significantly different from BSA-LG; £*P* < 0.05 significantly different from OPL-LG.

Hyperglycemia, hyperinsulinemia, and ER stress have all been implicated in the development of NAFLD; it is plausible that hyperglycemia and hyperinsulinemia precede ER stress. Therefore, we investigated the effect of glucose concentration on insulin signaling and ER stress. Under LG conditions, the presence of insulin elevated Akt Ser^473^ phosphorylation (Fig. 7, *A* and *B*). Basal Akt phosphorylation was significantly higher in HG compared with LG conditions; insulin-induced phosphorylation of Akt was blunted in HG (Fig. 7, *A* and *B*). No change in total Akt was seen under any of the conditions (Fig. 7, *A* and *C*). OPL treatment of cells had no effect on Akt phosphorylation in either glucose condition (Fig. 7, *A* and *B*). HG significantly increased CHOP expression compared with LG levels (Fig. 7, *A* and *D*); CHOP expression was unaffected by insulin or lipid in both glucose conditions.

#### Effect of exogenous fatty acids and glucose concentration on AMPK signaling and oxidation in LIV0APOLY cells.

**Fig. 8.**
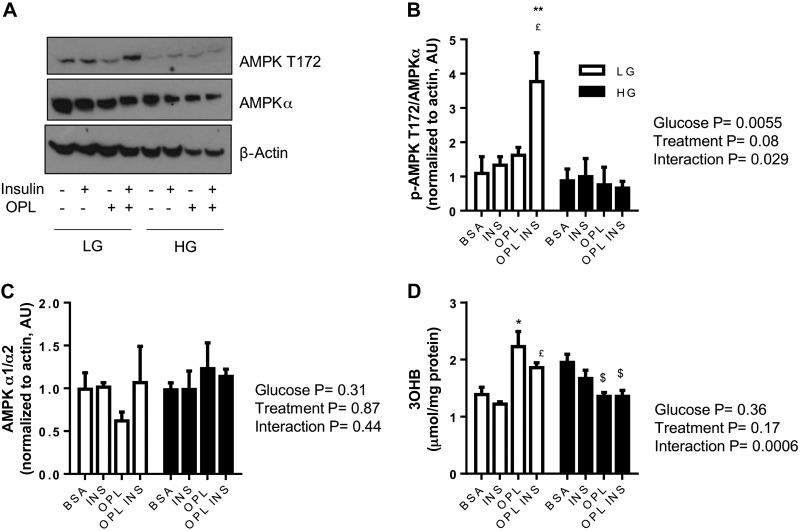
Effect of glucose and exogenous fatty acids on markers of fatty acid oxidation in LIV0APOLY cells. Cells were cultured under LG (5.5 mM) or HG (22.5 mM) and treated with insulin (100 nM) in the absence or presence of OPL for 24 h. *A*: immunoblots of total and Thr^172^ AMPKα, equal gel loading ascertained by immunoblotting against β-actin. Expressions of Thr^172^ (*B*) and AMPKα (*C*) were quantified relative to β-actin (*n* = 3) and expressed as AU. *D*: 3-hydroxybutyrate (3-OHB) secretion from LIV0APOLY cells. Data are means ± SE (*n* = 3). *P < 0.05; **P < 0.01, significantly different from BSA-LG; £*P* < 0.05 significantly different from LG-INS; $*P* < 0.05 significantly different from HG-BSA.

AMPK was significantly phosphorylated under conditions of LG in the presence of fatty acid (OPL) and insulin; an effect not observed under HG conditions (Fig. 8, *A* and *B*). Changes in AMPK phosphorylation were not due to altered expression of total AMPKα (Fig. 8, *A* and *C*). As a marker of fatty acid oxidation, we measured the appearance of 3-OHB in the medium. LIV0APOLY cells cultured in LG and OPL in the absence or presence of insulin had significantly elevated 3-OHB levels in the medium; the opposite effect was observed in HG (Fig. 8*D*).

#### Comparison of fatty acid partitioning in LIV0APOLY and human primary hepatocytes.

**Fig. 9.**
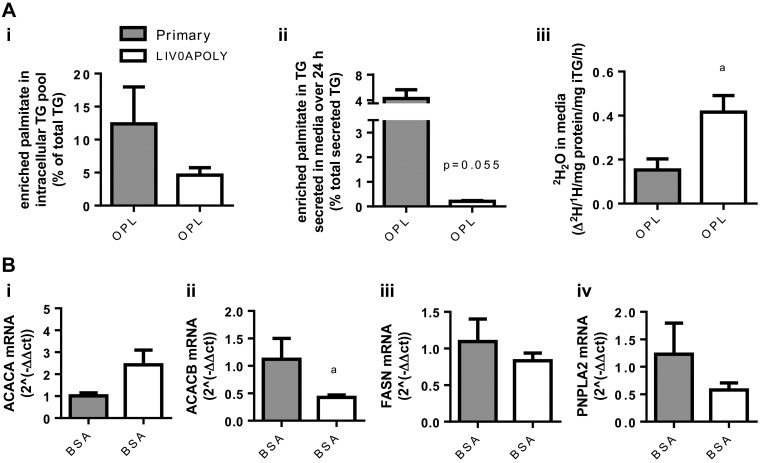
Fatty acid partitioning and gene expression in human primary hepatocytes and LIV0APOLY cells. LIV0APOLY (5.5 mM glucose) and primary hepatocytes were grown in the absence or presence of OPL for 24 h. *A*: percentage of enriched palmitate in *i*) intracellular TG pool, *ii*) secreted TG, and *iii*) quantification of ^2^H_2_O in media. *C*: mRNA expressions of *i*) *ACACA, ii*) *ACACB, iii*) *FASN*, and *iv*) *PNPLA2*. Housekeeping genes used were *HPRT1* and *GAPDH*. Data are means ± SE (*n* = 3). ^a^*P* < 0.05 primary human hepatocytes vs. LIV0APOLY cells.

To establish the usefulness of LIV0APOLY cells to investigate human NAFLD, we compared fatty acid partitioning in LIV0APOLY cells and isolated primary human hepatocytes (24 h after isolation). Both LIV0APOLY cells and human hepatocytes were incubated with OPL to investigate fatty acid partitioning, and to trace the fate of the exogenous fat, [D_31_]- and [U-^13^C]palmitate were utilized. There was no significant difference in the percentage of labeled palmitate incorporated into the intracellular TG pool in LIV0APOLY cells compared with primary human hepatocytes (Fig. 9*A*, *i*). However, the appearance of labeled TG in the medium from LIV0APOLY cells secreted less than 1% of labeled exogenous palmitate, whereas for primary human hepatocytes it was ∼ 4% (Fig. 9*A*, *ii*). We measured the appearance of ^2^H_2_O in the medium of LIV0APOLY and primary hepatocytes as a marker of fatty acid oxidation (29, 51); there was a significantly higher appearance of ^2^H_2_O (from [D_31_]palmitate) in the medium from LIV0APOLY cells than primary human hepatocytes (Fig. 9*A*, *iii*). A number of lipid metabolism genes were also measured by qPCR in LIV0APOLY and human primary hepatocytes under basal (BSA) conditions (after 24 h) to determine the presence or absence of the gene of interest without the effects of exogenous fatty acid supplementation. LIV0APOLY cells expressed comparable mRNA levels of *PNPLA2* (ATGL), *FASN* (FAS), and *ACACA* (ACC1) to that of primary human hepatocytes (Fig. 9*B*). Although LIV0APOLY cells expressed measurable amounts of *ACACB* (ACC2), expression was significantly lower than that of primary hepatocytes (Fig. *9B*).

## DISCUSSION

Cellular models that are more representative of fatty acid partitioning within the human liver are required to help elucidate the causes and progression of NAFLD. Ideally, human primary hepatocytes would be utilized; however, these come with limitations, including availability, culturing times, the effect of isolation on function, terminal differentiation, and lack of robust cryopreservation techniques. Therefore, we set out to characterize a novel human liver cell line that was able to proliferate and that expressed a hepatocyte-like phenotype, in order to establish a model of TG accumulation by using exogenous metabolic substrates such as fatty acids and glucose.

In the present work, human fetal liver cells were conditionally immortalized using c-mycER^TAM^ technology, which has been shown to enhance proliferation in a variety of cell types (31, 42). This resulted in a hepatocyte cell line (LIV0APOLY) whose proliferation could be driven by the presence of 4-OHT to culture medium. Standard cryopreservation (FBS, 10% DMSO) of LIV0APOLY cells had no effect on the doubling time (or viability) of the cells; in contrast, primary hepatocytes are reported to be poorly resistant to cryopreservation, which affects cell viability, morphology, and functionality (21). We found that the LIV0APOLY cell line expressed defined hepatocyte markers, such as albumin and CK18 (22, 26, 32, 54). Expression of albumin in two-dimensionally cultured primary hepatocytes has been shown to decrease after approximately 1 wk, whereas cultured LIV0APOLY cells retained high expression of albumin over 45 days and for at least 14 days following differentiation. Urea secretion increased significantly in differentiated LIV0APOLY cells, whereas long-term removal of 4-OHT (more than 7 days) resulted in a significant loss in CK18 expression and an increase in AFP expression. The expression of AFP+ cells in adult liver is reported to be 0.01% except in livers with severe injury or disease, where expression is increased (1, 27, 46). The increase in AFP protein observed here after 7 days of 4-OHT removal may have been due to cell damage or death. Cleaved CK18 in plasma is suggested to be a biomarker for liver fibrosis and apoptosis in humans (28); we found CK18 was undetectable in the culture medium from LIV0APOLY cells. Taken together, these data demonstrate that the LIV0APOLY cells, cultured for up to 5 days after initiation of differentiation, display a phenotype consistent with adult human hepatocytes. However, it should be noted that the expression of some cytochrome *P*-450 enzymes were induced by differentiation of LIV0APOLY cells at early passages (*CYP3A4*, *2D6*, and *2E1*; data not shown), while other genes were undetectable or very lowly expressed compared with human hepatocytes, e.g., *CYP7A1* and *FABP1*. Therefore, it would be prudent to suggest that phenotyping of cells, for the specific genes of interest, should be undertaken prior to their use in in vitro cellular studies.

It is well recognized that cells from healthy human livers contain lower amounts of intracellular TG compared with HepG2 cells (14, 52). This observation may be partly explained by HepG2 cells lacking the required machinery to secrete TG-rich lipoproteins (34, 48). In line with this, we found negligible amounts of TG in the medium from HepG2 compared with LIV0APOLY cells, which may have been due to a lower sensitivity in the methodology used to determine TG concentrations. Genotype may also play a role; for example, the hepatocellular carcinoma cell line Huh7/7.5 carries the *PNPLA3* rs738409 (Ile^148^Met) variant (3). It could be hypothesized that cells that carry the variant (either CG or GG) may display different metabolic properties, such as greater fatty acid uptake, lower TG turnover, less fatty acid oxidation, or impaired TG secretion. We report here that HepG2 cells express the variant *PNPLA3* genotype (GG), whereas LIV0APOLY cells express wild-type *PNPLA3* (CC). Therefore, it is plausible that the different genotype explains, in part, the higher intracellular TG content that we found in HepG2 compared with LIV0APOLY cells when cultured in the same concentration of glucose. To our knowledge, LIV0APOLY cells are the first reported wild-type *PNPLA3* hepatocyte cell line and therefore may be a useful cell model to investigate intracellular fatty acid partitioning independently of genotype effects.

To establish a model of hepatic TG accumulation, we treated cells with a physiological mixture of fatty acids, in a similar ratio to that most commonly observed in the systemic circulation of humans (24), in the absence or presence of insulin, thus mimicking, to some extent, the transition from a fasted to a fed state. In healthy humans, an increase in systemic insulin concentrations, such as in the postprandial period after consumption of a mixed test meal, has been reported to upregulate DNL (49); VLDL secretion is suppressed in response to insulin (33). Both insulin and HG significantly increased DGAT2 expression, which may reflect an increase in DNL; DGAT2 is required for the synthesis of de novo synthesized TG (55). HG also increased mRNA DGAT1 expression. DGAT1 has been shown to have bidirectionality, channeling TG into either secretion or storage pools (55). We assessed the concentration of TG in the medium as a marker of secretion and found it was not significantly elevated on HG compared with LG. Additionally, HG conditions increased CHOP expression, a marker of ER stress and a cell death trigger that has been suggested to play a role in the development of NAFLD (18).

Fatty acids within hepatocytes are broadly partitioned between esterification and oxidation pathways (23). To trace the fate of fatty acids in LIV0APOLY cells and primary human hepatocytes, we utilized stable isotope methodologies and found no difference in the relative contribution of labeled palmitate within intracellular TG of LIV0APOLY cells and primary human hepatocytes. However, we found a notably lower proportion of labeled TG in the medium of LIV0APOLY cells compared with primary human hepatocytes. As liver fat content has been associated with VLDL-TG production in humans (2), it is plausible that the amount of intracellular TG present in the cells prior to incubation with exogenous fatty acids may influence TG secretion. The primary human hepatocytes we cultured had been exposed to chemotherapy, which is known to increase liver fat content; we found these hepatocytes had a greater intracellular TG content than LIV0APOLY cells under basal conditions (data not shown). Gibbons et al. (19) reported that the exogenous fatty acids are not directly utilized to form VLDL; they first enter a temporary TG storage pool, which influences the rate at which VLDL-TG is secreted. The cells in the present study were incubated with fatty acids for 24 h, and it is possible that this was not a sufficient time period to see a marked increase in the appearance of TG in medium. An alternative explanation is that the machinery required for VLDL lipidation and secretion in LIV0APOLY cells is deficient under these conditions. Alternatively, a higher contribution of unlabeled palmitate from DNL may have led to dilution of the tracer in the medium, thus underestimating the relative contribution. We found that culturing LIV0APOLY cells in HG resulted in significantly more TG in the medium than LG, suggesting that LIV0APOLY cells preferentially secrete TG synthesized through the DNL pathway rather than from exogenous sources. Animal work has suggested that DNL fatty acids exit the liver immediately as VLDL-TG rather than being stored (15), although evidence in humans for this is sparse (50). There is evidence that the lipolysis-reesterification cycle supports VLDL secretion in primary human hepatocytes (30, 53). Hudgins et al. (25) reported a positive correlation between DNL and plasma TG levels in humans and that plasma TG increases proportionally to the amount of DNL-TG synthesized when on a low-fat diet.

An alternative route of disposal for fatty acids within the liver is oxidation. In LIV0APOLY cells grown in LG conditions we found that the medium concentrations of 3-OHB and phosphorylation of AMPK were increased when exogenous fatty acids were given in the absence or presence of insulin. Additionally, the appearance of ^2^H_2_O (from [D_31_]palmitate) was higher in the medium from LIV0APOLY cells compared with primary human hepatocytes. A possible reason for this discrepancy between cell types is a difference in culturing conditions; human primary hepatocytes are grown in medium containing 11 mM glucose and LIV0APOLY cells with 5.5 mM glucose, which may influence fatty acid partitioning. We found that culturing LIV0APOLY cells in HG compared with LG suppressed markers of fatty acid oxidation, in line with the notion that an upregulation of DNL attenuates fatty acid oxidation. This can be explained, as an increase in DNL would lead to an increase in malonyl-CoA, a potent inhibitor of carnitine palmitoyltransferase-1 (CPT1), the key enzyme responsible for transporting fatty acyl-CoA into the mitochondrion (23). Therefore, under conditions of excess glucose, cellular metabolism is shifted toward esterification rather than oxidation.

We demonstrate here that LIV0APOLY cells are a renewable and proliferative human hepatic cell line, that are wild type for the *PNPLA3* variant. Under basal conditions they do not have notable lipid accumulation, but they respond to short-term exposure of exogenous fatty acids by storing and oxidizing fatty acids. Although marginal, these cells secrete TG, and further work is required to determine their capacity for apoB-containing lipoprotein assembly and secretion. Additionally, they appear to dispose of excess glucose by intracellular fatty acid synthesis (DNL) to form TG, as would be predicted in vivo. Taken together, these cells can be considered a useful human liver cell model for studying the effects of exogenous metabolic substrates on fatty acid partitioning to allow future studies to delineate the mechanisms that may influence the development and progression of NAFLD.

## GRANTS

This project was funded by the OHSRC/BRC, Human Research Trust, and the British Heart Foundation (FS/11/18/28633). L. Hodson is British Heart Foundation Intermediate Fellow in Basic Science.

## DISCLOSURES

No conflicts of interest, financial or otherwise, are declared by the author(s). D.J., H.D.A., E.A.M., L.S., and J.D.S. are/were employees of ReNeuron, but the contents reflect only the authors' views and not the views of ReNeuron.

## AUTHOR CONTRIBUTIONS

Author contributions: C.J.G., M.A.S., L.M.W., E.A.M., J.D.S., K.J.M., and L.H. conception and design of research; C.J.G., D.J., H.D.A., P.S., L.S., and C.A.M. performed experiments; C.J.G., D.J., H.D.A., C.A.M., and E.A.M. analyzed data; C.J.G., E.A.M., J.D.S., K.J.M., and L.H. interpreted results of experiments; C.J.G. prepared figures; C.J.G. and L.H. drafted manuscript; C.J.G., D.J., H.D.A., P.S., M.A.S., L.M.W., L.S., C.A.M., E.A.M., J.D.S., K.J.M., and L.H. edited and revised manuscript; C.J.G., D.J., H.D.A., P.S., M.A.S., L.M.W., L.S., C.A.M., E.A.M., J.D.S., K.J.M., and L.H. approved final version of manuscript.
